# Co-culture with fibroblasts in stiff 3D scaffolds increases CD54 and CD140a expression on macrophages

**DOI:** 10.3389/fimmu.2026.1771248

**Published:** 2026-06-03

**Authors:** Jennessa WX Ng, Santosh TRB Rao, Emily H. Field, Kaitlyn Ritchie, Mark D. Wright, Nicholas P. Reynolds, Sean W. Cutter, Katrina J. Binger

**Affiliations:** 1 Department of Biochemistry & Chemistry, School of Agriculture, Biomedicine and Environment, La Trobe University, Melbourne, VIC, Australia; 2 Centre for Cardiovascular Biology & Disease Research, La Trobe Institute for Molecular Science (LIMS), La Trobe University, Melbourne, VIC, Australia; 3 Holsworth Biomedical Research Centre, Department of Rural Clinical Sciences, La Trobe Rural Health School, La Trobe University, Bendigo, VIC, Australia; 4 Department of Biology, School of Science, RMIT University, Melbourne, VIC, Australia; 5 Aikenhead Centre for Medical Discovery, Fitzroy, VIC, Australia; 6 Department of Immunology & Pathology, Alfred Medical Research and Education Precinct, School of Translational Medicine, Monash University, Melbourne, VIC, Australia; 7 Department of Biochemistry & Molecular Biology, Biomedicine Discovery Institute, Monash University, Clayton, VIC, Australia

**Keywords:** 3D culture, co-culture, macrophage activation, myofibroblast, vascular fibrosis

## Abstract

Vascular fibrosis is a major contributor to hypertension and cardiovascular disease and is caused by excessive extracellular matrix deposition and tissue stiffening. Macrophages and fibroblasts are key regulators of this process, yet most studies have relied on non-physiological two-dimensional (2D) *in vitro* culture systems, limiting their relevance. In this study, three-dimensional (3D) collagen-based scaffolds with tunable stiffnesses were developed to model the effect of fibrotic microenvironments on macrophage and fibroblast interactions. Bone-marrow derived macrophages (BMDMs) and mouse embryonic fibroblasts (MEFs) co-cultured in 3D maintained equivalent viability in scaffolds with a storage modulus of ~200 Pa and ~2000 Pa. Strikingly, during co-culture, macrophages, but not fibroblasts, exhibited a stiffness-dependent upregulation of CD54 (ICAM-1) and CD140a (PDGFRα), markers associated with inflammation and fibrosis. Confocal imaging revealed only occasional direct interaction between cell types, suggesting that the altered macrophage phenotype is driven by combined mechanical and soluble cues rather than increased physical interaction. Overall, these findings highlight the importance of dimensionality and stiffness in shaping macrophage-fibroblast crosstalk and provide a platform for dissecting mechanisms underlying vascular fibrosis.

## 1 Introduction

Vascular fibrosis both initiates and amplifies hypertension, contributing to cardiovascular complications and increased mortality (1, 2). Mechanical stress from high blood pressure activates cells including macrophages, fibroblasts, endothelial cells, and vascular smooth muscle cells to restore vessel homeostasis (3). Macrophages are innate immune cells important for inflammation, tissue repair and wound healing (4). While macrophages typically adopt different phenotypes in response to pathogens or cytokines (4), their function is also influenced by growth factors (5), mechanical stiffness (6), and extracellular matrix (ECM) proteins (7). Aberrant macrophage activation and accumulation within vessel walls is central to the development of fibrosis (8, 9). Once present, activated macrophages stimulate fibroblast proliferation and myofibroblast differentiation, increasing the deposition of ECM proteins such as collagen, leading to vessel rigidity (10). This increased stiffness raises vascular resistance, as arteries are unable to expand to accommodate blood flow, further elevating blood pressure. The resulting cycle of vessel damage, wound healing responses, and further macrophage and fibroblast activation creates a pathological feedback loop that worsens hypertension rather than restoring homeostasis, ultimately leading to a downward spiral of end-organ damage and mortality (3, 11).

The interplay between macrophages and fibroblasts and their precise contribution to the initiation and amplification of vascular fibrosis remains unclear. Although both cells drive fibrosis, they are also essential for resolving damage and restoring homeostasis, making therapeutic intervention difficult (12). Recent studies suggest these cells form a “cell circuit” *via* growth factor exchange that promote mutual proliferation and survival, including through direct contact (13). However, most findings derive from traditional two-dimensional (2D) cultures, which are orders of magnitude stiffer than human tissues. While plastic dishes exhibit a storage modulus of ~10^9^ Pa, healthy tissue ranges from ~100–1000 Pa in soft organs like the liver, up to ~20,000 Pa in stiffer organs like the heart (14). While macroscopic vascular tissue reaches ~10^6^ Pa, individual cells within healthy vessel walls sense a microscopic stiffness of ~1,000-100,000 Pa (15, 16). Substrate stiffness strongly influences stem cell fate: for example, culturing stem cells on stiff gels mimicking bone enhances osteogenesis (17), while cardiomyocytes beat most efficiently on gels with similar stiffness to the heart (18). Yet, how mechanical cues modulate the function of already differentiated cells such as macrophages and fibroblasts is unclear. Previous studies show that fibroblast-to-myofibroblast differentiation in 3D collagen-alginate scaffolds is promoted by increased stiffness (19), and monocyte differentiation toward macrophage pro-fibrotic phenotypes is influenced by 3D co-culture with pancreatic cancer cells and cancer-associated fibroblasts (20). These observations are relevant to fibrosis, which increases tissue stiffness by ~10-20-fold, altering cellular function, restricting blood flow and impairing organ mechanics (21–23).

In this study, a 3D co-culture system with tuneable stiffness was developed to investigate how mechanical cues and cell-cell interactions shape macrophage and fibroblast phenotypes. Co-culturing these cells in 3D scaffolds with a 10-fold increased stiffness increased CD54 (intercellular adhesion molecule 1, ICAM-1) and CD140a (platelet-derived growth factor receptor alpha, PDGRFα) expression on macrophages. These findings highlight the importance of mechanical and cellular contexts in regulating immune-stromal crosstalk and provide a platform for further dissecting mechanisms underlying vascular fibrosis.

## 2 Methods

### 2.1 Culture of bone-marrow derived macrophages and mouse embryonic fibroblasts

Procedures were approved by the La Trobe University Animal Ethics Committee. Bone-marrow derived macrophages (BMDMs) were generated from bone marrow cells derived from wildtype C57Bl/6 male mice (6–12 weeks old) and differentiated into macrophages by culture for 7 days with 10 ng/mL recombinant murine colony stimulating factor (M-CSF) (312-02, PeproTech), as described previously (24). Mouse embryonic fibroblasts (MEFs) were immortalized by infection with a Lentivirus encoding the large T SV40 (25) and were a kind gift from Professor Ivan Poon (La Trobe University, Australia). Both BMDMs and MEFs were cultivated in RPMI-1640 culture media supplemented with 10% FBS, 2 mM L-Glutamine, and 1% penicillin/streptomycin at 37 °C, 5% CO_2_. No M-CSF was present during subsequent mono- or co-cultures of BMDMs and MEFs.

### 2.2 Cell culture in 3D scaffolds and 2D culture plates

Two formulations of collagen-based 3D scaffolds were utilized: collagen-vitronectin and collagen-vitronectin-alginate. Soluble preparations containing the desired ECM proteins and cells were mixed, followed by collagen gelation at 37 °C for 1 h, followed by the addition of 50 mM CaCl_2_ for 5 min to induce crosslinking of alginate. Briefly, alginate stock solutions (2x) were prepared by solubilizing solid sodium alginate into RPMI-1640 culture media containing BMDMs and/or MEFs. These solutions were mixed 1:1 on ice with a soluble formulation of bovine Type I collagen (5074, 5 mg/mL, PureCol^®^, Advanced Biomatrix). Vitronectin (10424-H08H, SinoBiological) was added to a final concentration of 5 µg/mL. Final concentrations within the scaffolds were 2.5 mg/mL collagen, 1% w/v, 5% w/v or 10% w/v alginate, and 5 µg/mL vitronectin. BMDMs and MEFs were cultured for 24 h or 6 days. For all 24 h experiments, a consistent density of 1 x 10^6^ cells/mL was maintained across all conditions, resulting in 150,000 cells/sample when cultivated in 96-well plates. In co-culture experiments, BMDMs and MEFs were mixed at a 1:1 ratio (0.5 x 10^6^ cells/mL each), maintaining a total density of 150,000 cells/96-well (75,000 cells of each population). For longitudinal (6 day) experiments conducted in 96-well plates, the initial seeding density of MEFs was reduced to 20,000 cells/well to permit expected proliferation in 2D. In these same longitudinal experiments, non-proliferative primary BMDMs were seeded at 150,000 cells/well to ensure adequate signal detection. Following collagen gelation and alginate cross-linking, excess calcium chloride was removed by two washes with PBS. Additional culture media was then added to ensure hydration of the gels. 2D controls were prepared by coating non-treated plates with a solution of 0.5 mg/mL collagen in 0.02 M acetic acid for 2 hours at 37 °C, followed by two PBS washes. To ensure parity, 2D cultures were seeded at the same densities and media volumes as 3D scaffolds. All conditions underwent identical incubation, CaCl_2_ treatment and washes. Where indicated, BMDM monocultures were activated with 10 ng/mL lipopolysaccharide (LPS; tlrl-3pelps, Invivogen) and 10 ng/mL murine interferon gamma (IFNγ; Abcam), or 10 ng/mL murine interleukin (IL-4; PeproTech) and 10 ng/mL IL-13 (PeproTech), as indicated. Cultures were incubated at 37 °C, 5% CO_2_ for the specified durations.

### 2.3 Rheology

The stiffness of cell-free 3D scaffolds was measured by rheology using an Anton Paar MCR-302 rheometer (Anton Paar) equipped with a 10 mm parallel plate (PP10 RFID) (Anton Paar) as described previously (26). Collagen-based 3D scaffolds containing collagen-alone, collagen-vitronectin, or collagen-vitronectin-alginate were prepared as described above. Amplitude sweeps were carried out at 37°C with a constant applied force of 0.05 N to measure the storage (G’) and loss (G”) modulus of the gels between 0.001% and 100% shear strain at an angular frequency of 10 rads^-1^. The linear viscoelastic region (LVR) of the gels was identified as the region in which the storage modulus remained constant with increasing shear strain; for all formulations, this occurred between approximately 0.01-0.2% shear strain. A shear strain of 0.1% was selected as a representative value within the LVR for all materials, and the storage modulus at this value was reported as the material stiffness (Pa).

### 2.4 AlamarBlue assays

AlamarBlue (DAL1100, Invitrogen) assays were used to measure the *in situ* metabolic activity of 2D and 3D cultured cells. This assay quantifies the reduction of resazurin to resorufin by cellular reducing agents (e.g., NADH, NADPH, and FADH_2_) produced during cellular metabolism. For all experimental samples, 25 μL of alamarBlue reagent was added to each well and cells were incubated for 2 hours at 37°C and 5% CO_2_ according to the manufacturer’s protocols. Fluorescence was measured with excitation and emission of 560 nm and 590 nm, respectively, on a SpectraMax M5e plate reader (Molecular Devices). To validate the relationship between cellular metabolism and cell density, a 2D calibration assay was performed. BMDMs and MEFs were seeded into 2D plasticware at increasing densities (0 – 150,000 cells/well) and allowed to adhere before measuring alamarBlue fluorescence to confirm a linear relationship (**Supplementary Figures 1A, B**). Generating a 3D calibration curve was not possible due to difficulties with ensuring uniform cell seeding and consistent nutrient diffusion across a wide range of seeding densities. Thus, relative (or normalized) alamarBlue fluorescence was calculated by dividing the signal of each sample by the mean signal of its equivalent samples at *t* = 0 h. Absolute alamarBlue fluorescence was also calculated by subtracting readings from cell-free negative controls from all values. For 2D samples, this consisted of collagen-coated wells containing cell media, and for 3D cell-free scaffolds were prepared as described above.

### 2.5 Flow cytometry

BMDMs or MEFs were extracted from 3D cultures by enzymatic digestion. Samples were first incubated with 1.6 mg/mL collagenase (Type IV; 17104019, ThermoFisher) for 20 minutes at 37 °C in a multitron incubator shaker (Infors HT), followed by 20 mg/mL alginate lyase (A1603, Sigma-Aldrich) for another 15 minutes at 37 °C in a multitron incubator shaker. Gentle pipetting with additional PBS supplemented with 0.5% v/v FBS, 2 mM EDTA was performed to break down any remnant 3D scaffold. 2D cells were similarly treated with enzymes, then detached from plastic plates by incubation with PBS supplemented with 0.5% v/v FBS, 2 mM EDTA for 10 minutes at 37°C, followed by gentle pipetting. The resultant single cell suspensions were then pelleted at 400 g for 5 min, and resuspended in fixable viability dye FV780 (65-0865-18, eBiosciences) and incubated on ice for 30 minutes. Samples were then centrifuged and resuspended in Fc receptor blocking antibodies (1:50 CD16/CD32; 14-0161-82, eBioscience) in FACS buffer (PBS with 0.5% FBS, 2 mM EDTA) and incubated for 10 minutes on ice. The desired antibodies were then added and samples were incubated for a further 15 minutes on ice. Antibodies included: F4/80-e450 (1:400; #48-4801-82, eBioscience™), CD45.2-e506 (1:200; #69-0454-82, eBioscience™), CD140a-BV711 (1:100; #740740, BD Biosciences), CD11b-FITC (1:400; #11-0112–85 eBioscience™), CD54-PE (1:200; #12-0541-82, eBioscience™). Samples were then washed once with FACS buffer and analyzed by BD FACSymphony™ A3 Cell Analyzer (BD Biosciences). Data were analyzed using FlowJo V10 software (BD Biosciences).

### 2.6 Microscopy

BMDMs were labelled with CellTrace Far-Red DDAO-SE (1 μg/mL; C34553, Invitrogen) and MEFs with CellTracker Green CMFDA (1 μg/mL; C7025, Invitrogen). Dyes were prepared as per the manufacturer’s instructions and single cell suspensions were labelled by incubation with their respective dyes for 30 minutes at 37°C, before washing thrice with PBS. Fluorescently labelled BMDMs and MEFs were then mixed 1:1 (total cell concentration 0.75 x10^6^ cells/mL) and seeded into 3D collagen-vitronectin and 3D collagen-vitronectin-alginate scaffolds prepared within µ-Slide 8-well high chamber slides (#80807, Ibidi) for confocal microscopy. Cells were co-cultured overnight at 37°C, 5% CO_2_ before the addition of Hoechst 33342 (1 μg/mL; 14533, Thermo Fisher) for 10 minutes at room temperature to stain nuclei. Cells were then washed thrice with PBS. Samples were imaged using an Andor Dragonfly spinning disk confocal microscope (Oxford Instruments). Images were processed on ImageJ imaging software.

### 2.7 Statistical analysis

All statistical analysis was performed using GraphPad Prism software. Experiments were performed at least three times independently with pooled means represented as individual data points with error bars representing the standard error of the mean (SEM). For one-way ANOVA, data was first tested for normality using a Shapiro-Wilk test and then analyzed by an ordinary one-way ANOVA or Kruskal Wallis test with Bonferroni or Dunn’s *post-hoc* analyses, respectively. For two-way ANOVA, *post-hoc* analyses were by Tukey. Specific tests are indicated in each figure legend.

## 3 Results

### 3.1 Designing a tunable 3D collagen-based scaffold to mimic fibrotic stiffness

**Figure 1:**
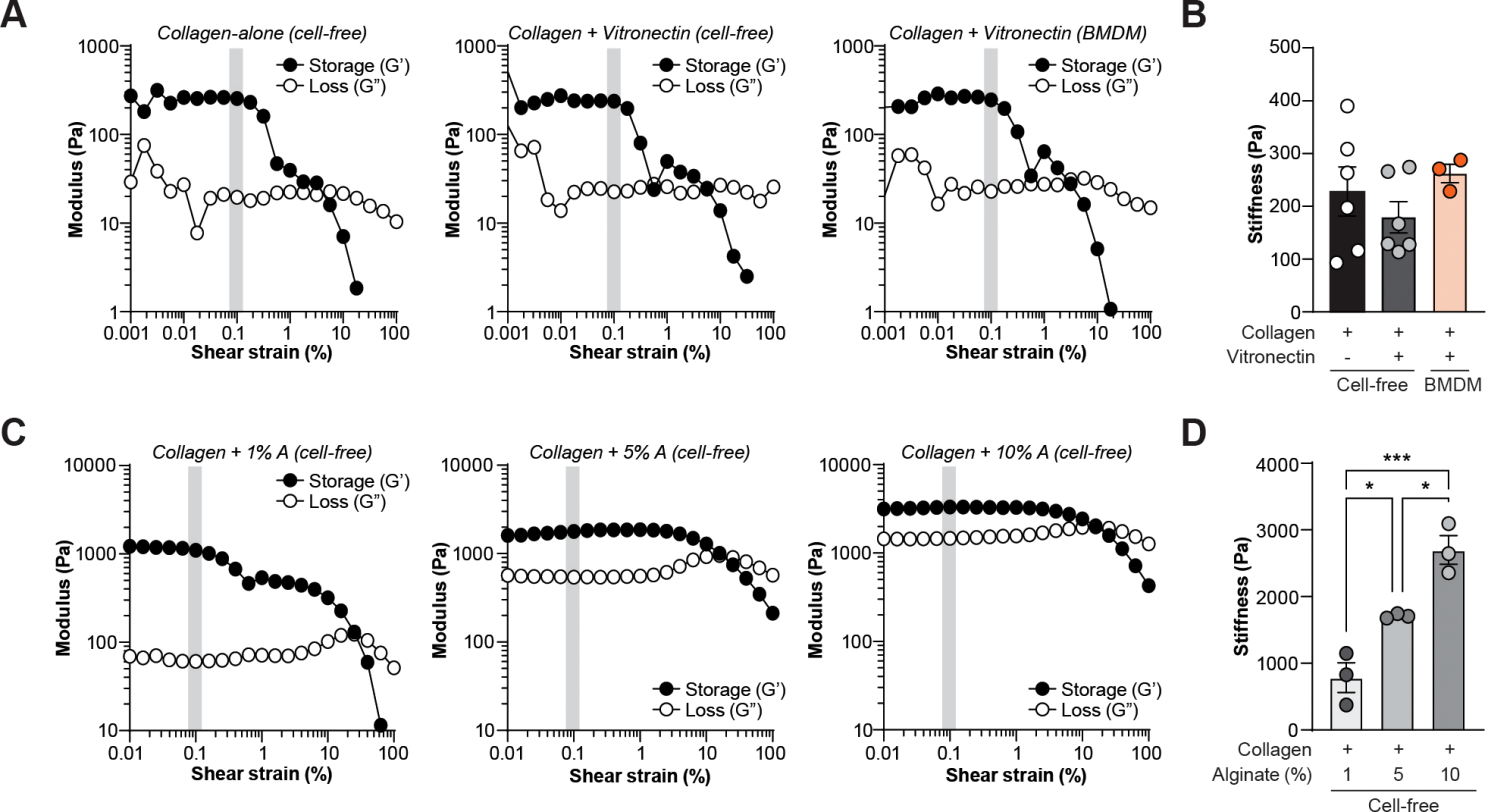
The addition of alginate increases 3D collagen-vitronectin scaffold stiffness. **(A)** The storage (G’) and loss (G”) moduli of cell-free collagen-only and collagen-vitronectin 3D scaffolds, and collagen-vitronectin scaffolds seeded with bone-marrow derived macrophages (BMDMs) was measured by rheology with increasing shear stress (%). The linear viscoelastic region (LVR) was examined where 0.1% (grey bar) was selected to compare the stiffness of the materials. **(B)** The stiffness of cell-free collagen and collagen-vitronectin 3D scaffolds, and collagen-vitronectin scaffolds seeded with bone-marrow derived macrophages (BMDMs) was calculated at a shear strain at 0.1%. **(C)** Alginate (A) was included at increasing concentration (% w/v) to 3D collagen scaffolds and their stiffness was determined by rheology with increasing shear stress (%) following 5 mins crosslinking with calcium chloride. As per panel A, a shear strain of 0.1% (grey bar) was selected to compare material stiffness. **(D)** The stiffness of collagen-alginate scaffolds with increasing alginate concentration (% w/v) at a shear strain of 0.1%. Data points in B and D are the means of independent experimental replicates and error bars show the standard deviation of mean (SEM). Significance in B and D was measured by one-way ANOVA with *post-hoc* analyses: *p<0.05, ***p<0.001.

3D *in vitro* cultures were created to partially recapitulate the dimensionality and increased stiffness encountered by macrophages and fibroblasts within the fibrotic vasculature. 3D scaffolds were generated using a commercial bovine collagen I solution and their biomechanical properties were characterized using rheology. Amplitude sweeps were performed, in which the 3D scaffolds were subjected to increasing deformation (shear strain: 0.001-100%) while monitoring the storage modulus (G’), representing elastic properties, and loss modulus (G”), reflecting viscous behavior (27). Collagen-only 3D scaffolds exhibited viscoelastic solid behavior characterized by G’ greater than G” (**Figure 1A**) with an average stiffness of ~200 Pa (corresponding to the G’ value within the LVR at 0.1% shear strain) (**Figure 1B**). In both biological and *in vitro* settings, macrophages and fibroblasts adhere to surfaces *via* integrin receptor recognition of protein motifs, with one of the most common being arginine-glycine-aspartate (RGD) (28). While collagen provides structural scaffolding, vitronectin, a 75 kDa soluble ECM glycoprotein containing surface-exposed RGD motifs, was introduced to facilitate broader integrin engagement. As serum vitronectin levels are associated with an increased risk of cardiovascular disease (29), its incorporation is relevant for replicating the biochemical complexity of a fibrotic vascular environment. The addition of vitronectin did not significantly affect scaffold stiffness under cell-free conditions or upon the inclusion of bone-marrow derived macrophages (BMDMs) (**Figures 1A, B**). Therefore, to increase scaffold stiffness, alginate was incorporated into the system, a polysaccharide widely used in 3D culture systems and biomedical applications due to its biocompatibility and relative inertness on cellular function (30). It also readily interacts with divalent cations, allowing the generation of hydrogels with tunable stiffness upon the addition of solutions such as calcium chloride (26, 31). Hybrid 3D collagen scaffolds with increasing alginate concentration were prepared, and amplitude sweeps were performed as before following collagen gelation and alginate crosslinking at shear strain of 0.01-100% (**Figure 1C**). Scaffold stiffness increased in correlation with alginate concentration; specifically, 5% (w/v) alginate produced a scaffold approximately 10-fold stiffer than collagen-only scaffolds (**Figure 1D**).

**Figure 2:**
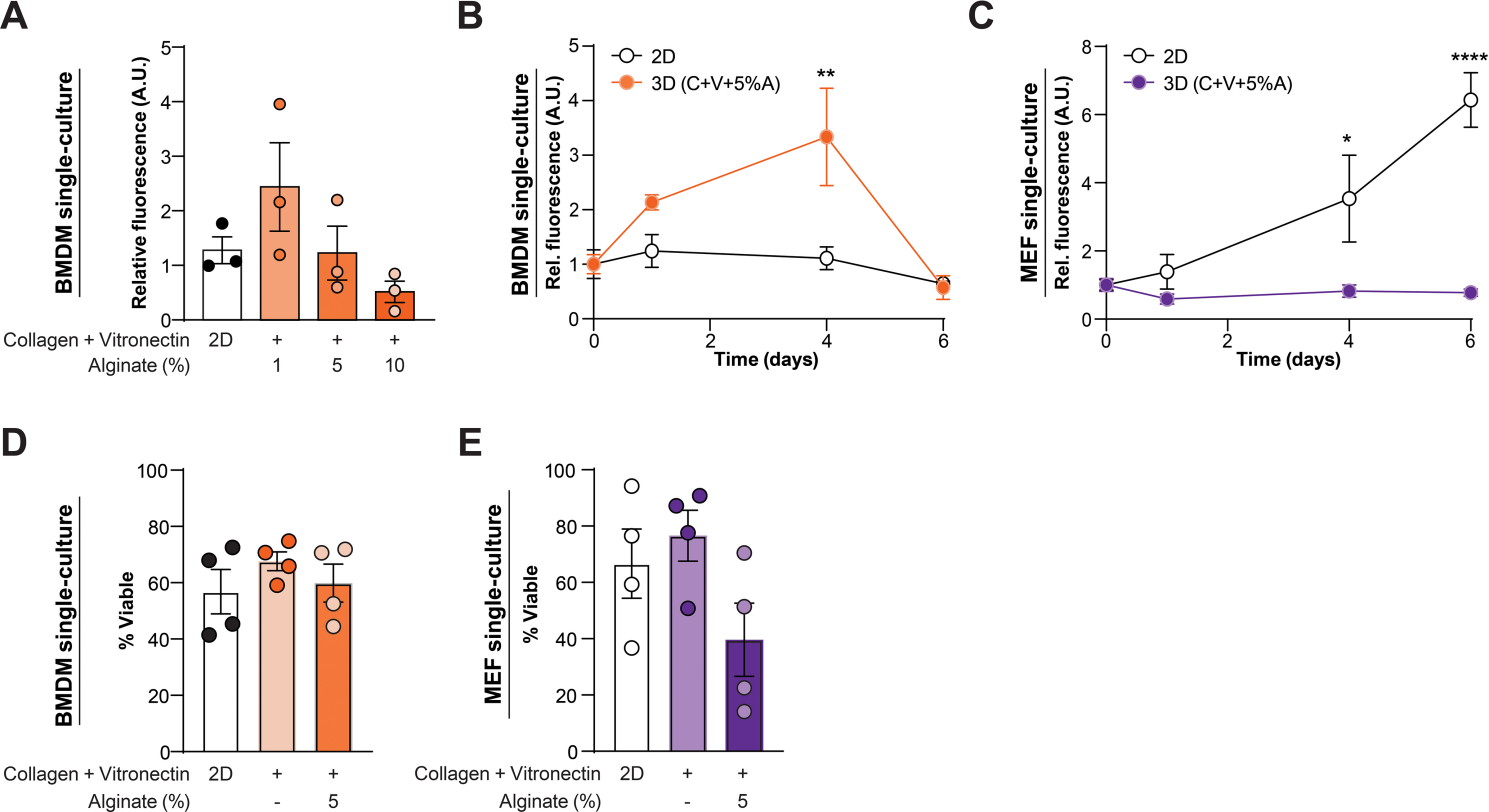
BMDMs and MEFs have equivalent viability upon culture in 3D collagen-vitronectin-alginate scaffolds. **(A)** BMDMs were seeded in 3D collagen-vitronectin scaffolds with increasing concentrations of alginate. Traditional 2D plasticware coated with a monolayer of collagen was compared as a control. Cells were cultured for 24 h before addition of alamarBlue. Relative fluorescence readings were calculated by dividing *t* = 24 h signals by cells cultured in respective 3D scaffolds or 2D controls at *t =* 0 h. **(B)** BMDMs and **(C)** MEFs were cultured separately in 3D collagen-vitronectin-5% alginate scaffolds or 2D controls for 6 days. The relative change in alamarBlue fluorescence was determined as in panel **(A)**. **(D)** BMDMs and **(E)** MEFs were cultured separately in 3D collagen-vitronectin, collagen-vitronectin-5% alginate scaffolds, or 2D controls for 24 h. Cells were isolated from 3D scaffolds by enzymatic digestion for analysis of cellular viability by flow cytometry. All experiments are the pooled means of at least 3 independent experiments with individual data points shown in **(A, D, E)**. Error bars show SEM. Significance in **(A, D, E)** was tested by one-way ANOVA; **(B, C)** by two-way ANOVA with *post hoc* analyses. Significance is indicated as *p<0.05, **p<0.01, ****p<0.0001.

Cell viability was next assessed using an *in situ* alamarBlue assay. After 24 hours culture on traditional 2D plasticware, BMDM fluorescence was ~3,000 units which significantly increased upon seeding within 3D 1% and 5% collagen-vitronectin-alginate scaffolds, suggesting an increased metabolic response upon 3D culture (**Supplementary Figure 1C**). To distinguish relative cell viability from this metabolic shift, data were normalized to *t* = 0 h controls. This analysis revealed that culture in scaffolds with differing alginate concentration did not affect relative alamarBlue fluorescence (**Figure 2A**). Metabolic activity and the relative viability of BMDMs and immortalized mouse embryonic fibroblasts (MEFs) cultured separately were compared over a longer duration in collagen-vitronectin-5% alginate scaffolds (**Figures 2B, C**; **Supplementary Figures 1D, E**). While 3D BMDMs maintained higher absolute alamarBlue fluorescence than 2D controls (**Supplementary Figure 1D**), normalized data showed comparable viability trends, with both culture environments resulting in a gradual decline in BMDM alamarBlue signal, typical of non-proliferative primary cells (**Figure 2B**). MEFs similarly exhibited an increased absolute alamarBlue fluorescence signal in 3D (**Supplementary Figure 1E**), with an opposite trend in relative cell viability with 2D cultures showing a time-dependent increase in normalized alamarBlue fluorescence, reflecting proliferative capacity, whereas a gradual decline was evident in 3D cultured MEFs (**Figure 2C**). Taken together, these results reveal distinct metabolic and proliferative responses of BMDMs and MEFs in 3D collagen-vitronectin-alginate scaffolds. To determine whether these changes reflected altered viability, flow cytometry was used to quantify viable cells. After 24 hours, BMDMs and MEFs maintained equivalent viability across all conditions (**Figures 2D, E**). Taken together, these data describe the generation of a composite collagen-vitronectin-alginate 3D scaffold with increased stiffness that supports viable culture of both BMDMs and MEFs.

### 3.2 Impact of scaffold stiffness on macrophage-fibroblast crosstalk

**Figure 3:**
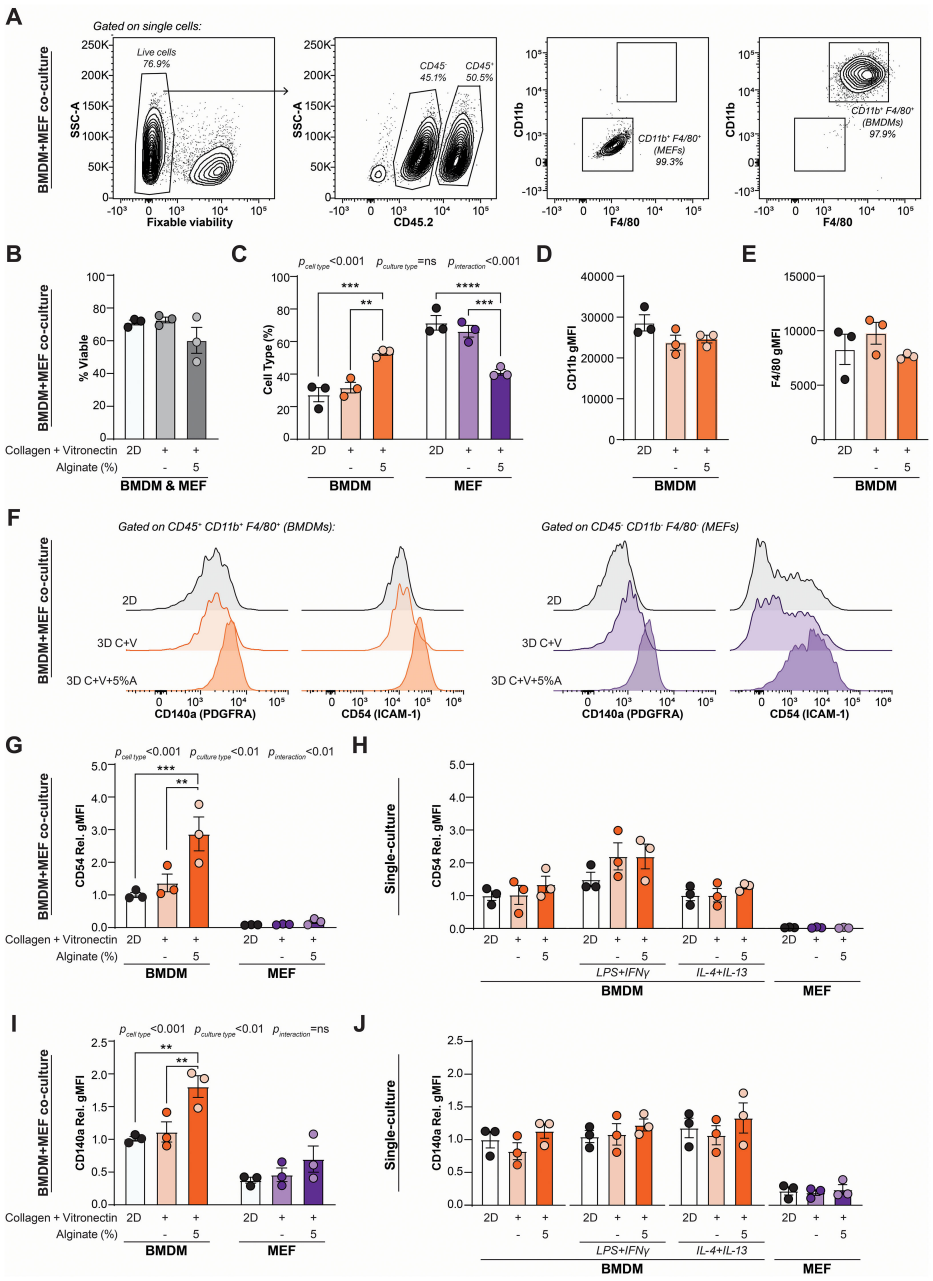
Co-culture of BMDMs and MEFs in stiff 3D hydrogels increases CD54 and CD140a expression on macrophages. BMDMs and MEFs were co-cultured in 3D collagen-vitronectin, collagen-vitronectin-5% alginate scaffolds, or 2D controls for 24 hours. The viability, proportion of each cell type and surface expression of key markers was determined by flow cytometry. **(A)** Representative gating strategy for the separation and analysis of co-cultured BMDMs and MEFs by flow cytometry. Sample shown are BMDMs and MEFs cultured in stiff collagen-vitronectin-5% alginate hydrogels. **(B)** Overall cell viability. Note that this represents a mixture of BMDMs and MEFs. **(C)** Proportion of BMDMs (CD45^+^CD11b^+^F4/80^+^) and MEFs (CD45^-^CD11b^-^F4/80^-^) in the indicated co-culture conditions. **(D, E)** Geometric mean fluorescence intensity (gMFI) of CD11b and F4/80 expression on co-cultured BMDMs. **(F)** Representative histograms for CD54 and CD140a expression on BMDMs (left) and MEFs (right). **(G–J)** The relative expression of CD54 **(G, H)** and CD140a **(I, J)** on BMDMs and MEFs after co-culture **(G, I)** or single-culture **(H–J)** in the indicated models. Some single-cultured BMDMs were also activated with LPS and IFN-γ or IL-4 and IL-13, as indicated. Data in G-J are the relative gMFI, calculated by dividing gMFI values by the mean gMFI of unactivated BMDMs cultured in 2D. B-E and G-J show the pooled means of 3–4 independent experiments. Errors are SEM. Groups in B, D, E, H and J were tested by one-way ANOVA; C, G and I by two-way ANOVA where for the latter overall ANOVA results are indicated above each graph. *Post-hoc* analyses were performed for one- and two-way ANOVAs where significance between groups is indicated as **p<0.01, ***p<0.001, ****p<0.0001, ns, not significant.

The 10-fold stiffness differential between “soft” 3D collagen-vitronectin and “stiff” 3D collagen-vitronectin-5% alginate scaffolds offered an opportunity to model the effect of tissue fibrosis on macrophage-fibroblast interactions. BMDMs and MEFs were co-cultured at a 1:1 ratio in soft and stiff 3D scaffolds and 2D controls for 24 hours. This duration was selected as cell viability remained equivalent across all culture conditions at this time point (**Figures 2D, E**). Following incubation, flow cytometry was utilized to analyze the populations, using a gating strategy to separate macrophages (CD45^+^F4/80^+^CD11b^+^) and fibroblasts (CD45^-^CD11b^-^F4/80^-^) (**Figure 3A**). While combined cell viability remained unchanged (**Figure 3B**), population proportions shifted. Despite seeding at a 1:1 ratio, BMDMs comprised only ~28% of the population in 2D cultures, which increased to ~53% in 3D collagen-vitronectin-5% alginate scaffolds (**Figure 3C**). Macrophage differentiation remained consistent between culture conditions, as indicated by unchanged expression of CD11b and F4/80 on BMDMs (**Figures 3D, E**). The equivalent proportion of BMDMs and MEFs in 3D therefore likely reflects robust MEF proliferation in 2D, which is suppressed in 3D (**Figure 2C**). The relative expression of CD54 (ICAM-1), a cell adhesion molecule that is induced on macrophages during inflammation and fibrosis, and CD140a (PDGFRα), a receptor for platelet-derived growth factor (PDGFRα) canonically associated with fibroblast proliferation and differentiation was next evaluated (**Figure 3F****;** **Supplementary Figure 2A**) (32–35). Strikingly, upon co-culture, CD54 exhibited a significant relative upregulation on BMDMs but not MEFs, with the degree of expression correlated with increasing 3D culture stiffness (**Figure 3G**; **Supplementary Figure 2B**). This relative induction was absent when BMDMs and MEFs were cultured separately, and was not observed following activation of BMDM monocultures into pro- and anti-inflammatory phenotypes (**Figure 3H**; **Supplementary Figure 2C**). Similarly, a significant relative upregulation of CD140a was observed on BMDMs co-cultured in stiff collagen-vitronectin-5% alginate 3D scaffolds (**Figure 3I**; **Supplementary Figure 2D**). This relative increase in CD140a was absent in 2D and 3D BMDM monocultures, regardless of activation state (**Figure 3J**; **Supplementary Figure 2E**). Notably, despite being a receptor normally expressed on fibroblasts, CD140a was not significantly increased on MEFs relative to baseline in any condition (**Figures 3I, J**).

**Figure 4:**
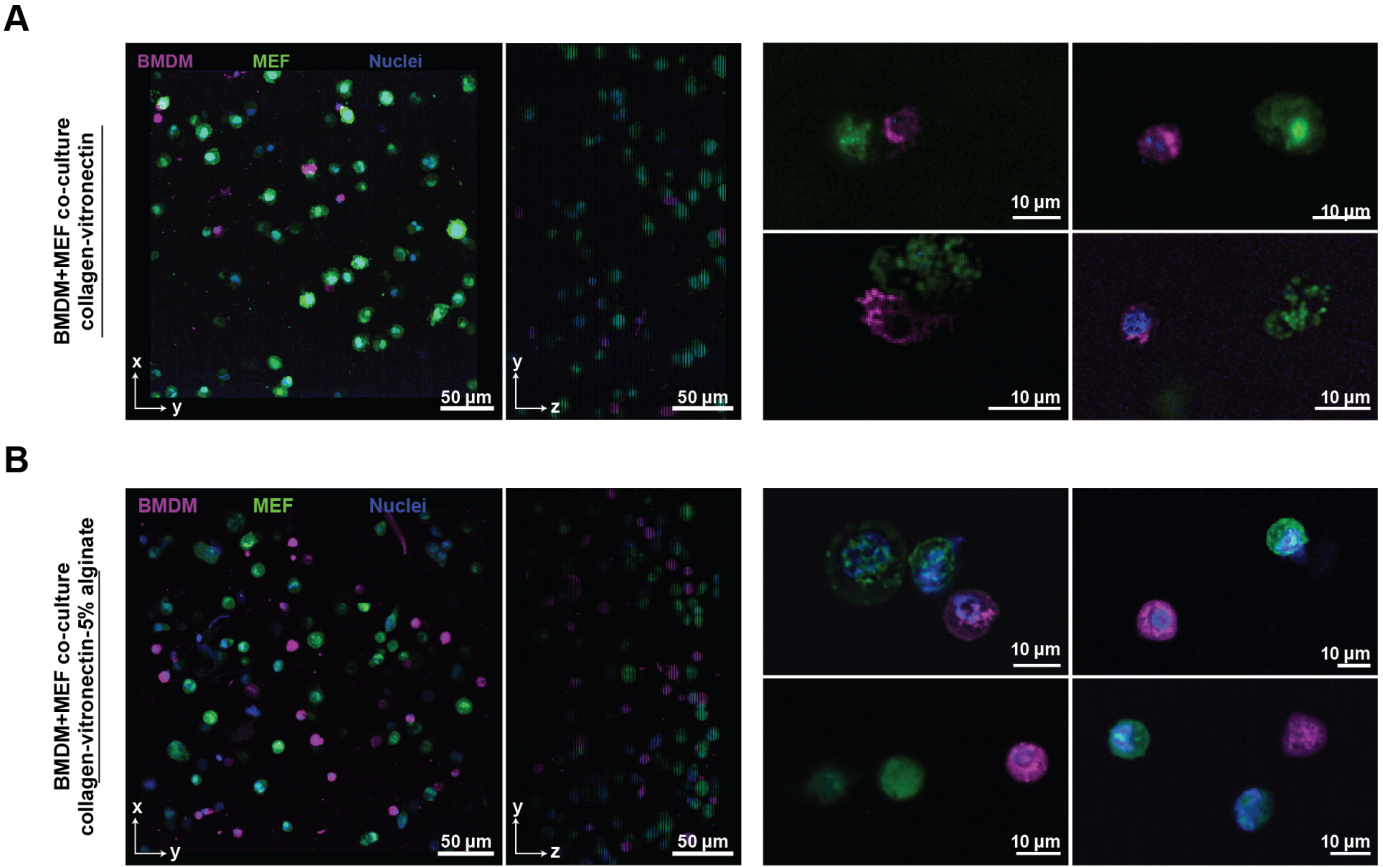
Spatial organization of BMDMs and MEFs in 3D co-culture. BMDMs and MEFs were labelled separately with the fluorescent dyes CellTrace Far-Red DDAO-SE and CellTracker Green CMFDA, respectively, and co-cultured in ‘soft’ 3D collagen-vitronectin **(A)** and ‘stiff’ 3D collagen-vitronectin-5% alginate **(B)** scaffolds for 24 hrs before visualization by spinning disc confocal microscopy. Right panels show examples of BMDM and MEF spatial organization at higher magnification. Scale bars are as indicated.

These findings suggest that stiffness, dimensionality, and co-culture with fibroblasts collectively drive a relative increase in the expression of CD54 and CD140a on macrophages. Spatial organization was visualized by spinning disc confocal microscopy. BMDMs and MEFs were stained separately with CellTrace Far-Red and CellTracker Green dyes, respectively, and co-cultured in collagen-vitronectin and collagen-vitronectin-5% alginate scaffolds for 24 hrs. Both BMDMs (red) and MEFs (green) were evenly distributed throughout both 3D scaffolds (**Figures 4A, B**). Instances of direct physical contact between BMDMs and MEFs were observed (**Figures 4A, B**, right panels), consistent with previous reports with 2D cultures (13, 36, 37). However, many cells remained isolated, and there was no apparent difference in spatial distribution between “soft” or “stiff” 3D scaffolds. Altogether, this indicates that the relative increase of CD54 and CD140a on macrophages in the stiffest 3D co-cultures is not correlated with increased physical interaction with fibroblasts.

## 4 Discussion

Macrophages and fibroblasts are present within all tissues and their proper function is essential for maintaining homeostasis. During fibrosis, the activation of both cell types results in excessive ECM deposition that ultimately stiffens tissues by approximately 10-20-fold (21–23). In this study, this pathological stiffening was modelled by generating hybrid 3D collagen-vitronectin-alginate scaffolds. It is demonstrated that co-culture with fibroblasts in these stiffer 3D microenvironments drives altered protein expression on macrophages, specifically of key molecules implicated in fibrotic processes. Notably, these changes were not evident in 2D culture, adding to a growing body of evidence that physiologically relevant 3D culture systems reveal aspects of cell biology that are hidden in traditional 2D culture conditions.

Recent studies have shown that fibroblasts and macrophages are highly sensitive to substrate stiffness. Both cell types sense mechanical cues *via* the transcriptional coactivator Yes-associated protein 1 (YAP1), a component of the Hippo-YAP1 pathway (5, 6). In fibroblasts, YAP1 was recently shown to regulate expression of colony-stimulating factor 1 (CSF-1), a cytokine critical for macrophage differentiation (5). In macrophages, increased activation of YAP1 and transcriptional coactivator with PDZ-binding motif (TAZ) enhances pro-inflammatory cytokine production, promoting a pro-inflammatory phenotype (6). In the present work, BMDMs exhibited a significant stiffness-dependent relative increase in CD54 expression when co-cultured with MEFs in 3D scaffolds, and this expression correlated with stiffness where collagen-vitronectin-5% alginate 3D cultures showed the highest expression (**Figure 3G**). CD54 (ICAM-1) facilitates macrophage efferocytosis, the phagocytosis of apoptotic and necrotic cells, and its expression is upregulated during inflammation (38). CD54 is also correlated with macrophage activation into a pro-inflammatory phenotype (39–41). Notably, this effect was absent when macrophages were cultured in 3D alone, either unactivated, or upon stimulation with LPS and IFN-γ. These findings suggest that the observed macrophage phenotype is not a response to stiffness alone, but by fibroblast-derived signals (likely soluble factors) that are themselves regulated by the mechanical and cellular environment. Such a mechanism is highly relevant to vascular fibrosis where increased vessel stiffness is sensed by fibroblasts, prompting their signaling to macrophages. In turn, macrophages may attempt to resolve tissue homeostasis *via* upregulation of molecules like CD54 to promote the clearance of cellular debris and enhance interactions with other immune cells. Although the absolute storage modulus of the collagen-vitronectin-5% alginate scaffolds (~2 kPa) is lower than the peak microscopic stiffness reported for fibrotic arteries, the 10-fold increase relative to collagen-vitronectin scaffolds (~0.2 kPa) accurately models the magnitude of the mechanical transition from a healthy to diseased vessel. While calcium chloride exposure was standardized across all conditions to eliminate it as a confounding variable, an inherent limitation of these hydrogels is that alginate concentration cannot be decoupled from stiffness. Because increasing alginate levels to achieve higher stiffnesses introduces preparation inconsistencies and restricts nutrient diffusion, exploring alternative materials that can be more precisely tuned to replicate fibrotic vascular stiffnesses remains an important objective for the continued development of this 3D system.

Although baseline CD140a expression is typically low, 3D co-culture drove a significant relative upregulation on BMDMs (**Figure 3I**), that correlated with 3D scaffold stiffness and the proportion of fibroblasts in co-culture. Crucially, this effect was marker-specific, as standard macrophage markers (CD11b, F4/80) remained static, confirming a targeted phenotypic shift rather than a generalized artefact of 3D co-culture. This unusual expression pattern may indicate the acquisition of fibroblast-like characteristics by macrophages. In fibroblasts, PDGFα-PDGFRα signaling is important in their early wound-healing response where *Pdgfra*-deficient fibroblast progenitors are less proliferative and do not differentiate into fibrosis-causing myofibroblasts (32). *In vitro*, fibroblasts cultured on 50 kPa substrates with interleukin-4 and GM-CSF showed an increased transition towards a myofibroblast phenotype compared to those cultured on 1 kPa environments (42). Moreover, 3D matrix interaction directly governs this differentiation. Fibroblasts permitted to spread in 3D alginate-collagen scaffolds *via* delayed calcium crosslinking exhibit enhanced myofibroblast differentiation compared to those forced into a more spherical morphology *via* immediate crosslinking (as utilized here) (43). Our findings demonstrate that interaction with the 3D environment also influences fibroblast-macrophage communication. Macrophage-to-myofibroblast transition (MMT) has been proposed as a mechanism contributing to fibrosis, mediated *via* activation of the transforming growth factor-beta (TGF-β) pathway (44). Following cardiac infarct, macrophage subpopulations have been identified that co-express PDGFRα and specific macrophage markers such as galectin-3 (Mac-2), CD107b/LAMP2 (Mac-3) and lysozyme-M (45). Due to the relative scarcity of cell-cell contact, it is speculated that the acquisition of PDGFRα on macrophages in stiff co-cultures is likely due to fibroblast-derived soluble factors; this may be in a similar mechanism to that previously identified for CSF-1, whose production by fibroblasts is regulated by mechanosensitive YAP signaling (5). An alternative explanation is the physical transfer of CD140a and other membrane components from fibroblasts to macrophages *via* trogocytosis. However, this mechanism would require frequent cell-cell contact between MEFs and BMDMs, which was observed only occasionally. Moreover, MEFs in our study expressed relatively low levels of CD140a, albeit with a modest increase upon 3D co-culture that did not reach significance.

Overall, these findings demonstrate that the combination of mechanical cues and cell-cell interactions within a 3D microenvironment drive altered macrophage phenotypes. Further characterization of the mechanisms underpinning this crosstalk between macrophages and fibroblasts in stiff 3D scaffolds may inform strategies for targeted drug development in fibrotic diseases.

## 5 Limitations

While this Brief Report highlights the importance of physiologically relevant culture systems, several limitations remain. First, the mechanisms underlying the relative upregulation of CD54 and CD140a on BMDMs during co-culture were not investigated. While treating cells with MEF-conditioned media is a logical next step, accurately modeling this secretome presents distinct technical challenges. Because mechanotransduction profoundly influences fibroblast behavior, 2D-derived conditioned media cannot faithfully recapitulate the 3D biochemical milieu; conversely, extracting soluble factors directly from 3D co-cultures risks cellular disruption. Future studies varying the ratio of macrophages:fibroblasts or modulating YAP activity will be important to assess functional consequences like efferocytosis or the acquisition of pro-fibrotic macrophage phenotypes. Second, this study relied on flow cytometry analysis of surface protein expression for this phenotypic characterization where future studies employing cell-sorting and transcriptomic profiling will be essential to define the molecular signature of both BMDMs and MEFs. Third, while the immortalized MEFs used here align with other *in vitro* co-culture studies, they may not fully capture primary myofibroblast behavior *in vivo.* Particularly, their differing 2D versus 3D proliferative capacities may mask stiffness-dependent effects on the fibroblasts themselves, despite profoundly altering the macrophage phenotype. Future studies utilizing modified encapsulation protocols to actively modulate fibroblast morphology, alongside employing pre-activated cells (e.g., with TGF-β), will be critical to interrogate the direct impact of the 3D environment on fibroblast activation, differentiation, and cross-talk to macrophages, thus improving the modelling of fibrosis more broadly. Fourth, because increased matrix stiffness was achieved *via* the incorporation of alginate, the differences observed during flow cytometry reflect both mechanical and compositional changes. Therefore, we cannot fully disentangle stiffness-driven effects from the potential biochemical contributions of the alginate polymer in this model, necessitating the future development of alternative tunable polymer systems. Finally, the analysis was limited to protein expression at a single, relatively short co-culture time point, where longer studies are required to determine if these macrophage phenotype changes persist, and/or whether the BMDMs and MEFs evolved into a self-sustaining “circuit”. Further to this would be studies modulating the ratio of BMDMs and MEFs will determine whether a certain threshold of fibroblasts are required to drive the relative upregulation of CD54 and CD140a.

## Data Availability

The raw data supporting the conclusions of this article will be made available by the authors, without undue reservation.

## References

[1] Harvey A Montezano AC Lopes RA Rios F Touyz RM . Vascular fibrosis in aging and hypertension: Molecular mechanisms and clinical implications. Can J Cardiol. (2016) 32:659–68. doi: 10.1016/j.cjca.2016.02.070. PMID: 27118293 PMC4906153

[2] Aune D Huang W Nie J Wang Y . Hypertension and the risk of all-cause and cause-specific mortality: An outcome-wide association study of 67 causes of death in the National Health Interview Survey. BioMed Res Int. (2021) 2021:9376134. doi: 10.1155/2021/9376134. PMID: 34337061 PMC8292050

[3] Humphrey JD . Mechanisms of vascular remodeling in hypertension. Am J Hypertens. (2021) 34:432–41. doi: 10.1093/ajh/hpaa195. PMID: 33245319 PMC8140657

[4] Wright MD Binger KJ . Macrophage heterogeneity and renin-angiotensin system disorders. Pflugers Arch. (2017) 469:445–54. doi: 10.1007/s00424-017-1940-z. PMID: 28176018

[5] Zhou X Franklin RA Adler M Carter TS Condiff E Adams TS . Microenvironmental sensing by fibroblasts controls macrophage population size. Proc Natl Acad Sci USA. (2022) 119:e2205360119. doi: 10.1073/pnas.2205360119. PMID: 35930670 PMC9371703

[6] Meli VS Atcha H Veerasubramanian PK Nagalla RR Luu TU Chen EY . YAP-mediated mechanotransduction tunes the macrophage inflammatory response. Sci Adv. (2020) 6:eabb8471. doi: 10.1126/sciadv.abb8471. PMID: 33277245 PMC7717914

[7] Cutter S Wright MD Reynolds NP Binger KJ . Towards using 3D cellular cultures to model the activation and diverse functions of macrophages. Biochem Soc Trans. (2023) 51 (1):387–401. doi: 10.1042/BST20221008. PMID: 36744644 PMC9987999

[8] Chan CT Moore JP Budzyn K Guida E Diep H Vinh A . Reversal of vascular macrophage accumulation and hypertension by a CCR2 antagonist in deoxycorticosterone/salt-treated mice. Hypertension. (2012) 60:1207–12. doi: 10.1161/HYPERTENSIONAHA.112.201251. PMID: 23033370

[9] Marko L Kvakan H Park JK Qadri F Spallek B Binger KJ . Interferon-gamma signaling inhibition ameliorates angiotensin II-induced cardiac damage. Hypertension. (2012) 60:1430–6. doi: 10.1161/HYPERTENSIONAHA.112.199265. PMID: 23108651

[10] Younesi FS Miller AE Barker TH Rossi FM Hinz B . Fibroblast and myofibroblast activation in normal tissue repair and fibrosis. Nat Rev Mol Cell Biol. (2024) 25:617–38. doi: 10.1038/s41580-024-00716-0. PMID: 38589640

[11] Froom ZS Callaghan NI Davenport Huyer L . Cellular crosstalk in fibrosis: Insights into macrophage and fibroblast dynamics. J Biol Chem. (2025) 301:110203. doi: 10.1016/j.jbc.2025.110203. PMID: 40334985 PMC12167814

[12] Buechler MB Fu W Turley SJ . Fibroblast-macrophage reciprocal interactions in health, fibrosis, and cancer. Immunity. (2021) 54:903–15. doi: 10.1016/j.immuni.2021.04.021. PMID: 33979587

[13] Zhou X Franklin RA Adler M Jacox JB Bailis W Shyer JA . Circuit design features of a stable two-cell system. Cell. (2018) 172:744–757.e17. doi: 10.1016/j.cell.2018.01.015. PMID: 29398113 PMC7377352

[14] Swift J Ivanovska IL Buxboim A Harada T Dingal PC Pinter J . Nuclear lamin-A scales with tissue stiffness and enhances matrix-directed differentiation. Science. (2013) 341:1240104. doi: 10.1126/science.1240104. PMID: 23990565 PMC3976548

[15] Ebrahimi AP . Mechanical properties of normal and diseased cerebrovascular system. J Vasc Interv Neurol. (2009) 2:155–62. PMC3317338 22518247

[16] Kohn JC Lampi MC Reinhart-King CA . Age-related vascular stiffening: causes and consequences. Front Genet. (2015) 6:112. doi: 10.3389/fgene.2015.00112. PMID: 25926844 PMC4396535

[17] Engler AJ Sen S Sweeney HL Discher DE . Matrix elasticity directs stem cell lineage specification. Cell. (2006) 126:677–89. doi: 10.1016/j.cell.2006.06.044. PMID: 16923388

[18] Engler AJ Carag-Krieger C Johnson CP Raab M Tang H-Y Speicher DW . Embryonic cardiomyocytes beat best on a matrix with heart-like elasticity: Scar-like rigidity inhibits beating. J Cell Sci. (2008) 121:3794–802. doi: 10.1242/jcs.029678. PMID: 18957515 PMC2740334

[19] Han Y Shao Z Zhang Y Zhao H Sun Z Yang C . 3D matrix stiffness modulation unveils cardiac fibroblast phenotypic switching. Sci Rep. (2024) 14:17015. doi: 10.1038/s41598-024-67646-x. PMID: 39043765 PMC11266583

[20] Kuen J Darowski D Kluge T Majety M . Pancreatic cancer cell/fibroblast co-culture induces M2 like macrophages that influence therapeutic response in a 3D model. PloS One. (2017) 12:e0182039. doi: 10.1371/journal.pone.0182039. PMID: 28750018 PMC5531481

[21] Foucher J Chanteloup E Vergniol J Castéra L Le Bail B Adhoute X . Diagnosis of cirrhosis by transient elastography (FibroScan): a prospective study. Gut. (2006) 55:403–8. doi: 10.1136/gut.2005.069153. PMID: 16020491 PMC1856085

[22] Aoyagi Y Schwartz AW Li Z Bai H Gonzalez L Lazcano Etchebarne C . Changes in vascular identity during vascular remodeling. JVS Vasc Sci. (2025) 6:100282. doi: 10.1016/j.jvssci.2025.100282. PMID: 40213096 PMC11985068

[23] Hinz B . Mechanical aspects of lung fibrosis: a spotlight on the myofibroblast. Proc Am Thorac Soc. (2012) 9:137–47. doi: 10.1513/pats.201202-017AW. PMID: 22802288

[24] McGowan EN Wong O Jones E Nguyen J Wee J Demaria MC . Tetraspanin CD82 restrains phagocyte migration but supports macrophage activation. iScience. (2022) 25:104520. doi: 10.1016/j.isci.2022.104520. PMID: 35754722 PMC9213772

[25] Poon IK Goodall KJ Phipps S Chow JD Pagler EB Andrews DM . Mice deficient in heparanase exhibit impaired dendritic cell migration and reduced airway inflammation. Eur J Immunol. (2014) 44:1016–30. doi: 10.1002/eji.201343645. PMID: 24532362

[26] Field EH Ratcliffe J Johnson CJ Binger KJ Reynolds NP . Self-healing, 3D printed bioinks from self-assembled peptide and alginate hybrid hydrogels. Biomater Adv. (2025) 169:214145. doi: 10.1016/j.bioadv.2024.214145. PMID: 39675342

[27] Janmey PA Georges PC Hvidt S . Basic rheology for biologists. Methods Cell Biol. (2007) 83:3–27. doi: 10.1016/S0091-679X(07)83001-9. PMID: 17613302

[28] Bachmann M Kukkurainen S Hytönen VP Wehrle-Haller B . Cell adhesion by integrins. Physiol Rev. (2019) 99:1655–99. doi: 10.1152/physrev.00036.2018. PMID: 31313981

[29] Derer W Barnathan ES Safak E Agarwal P Heidecke H Möckel M . Vitronectin concentrations predict risk in patients undergoing coronary stenting. Circ Cardiovasc Interv. (2009) 2:14–9. doi: 10.1161/CIRCINTERVENTIONS.108.795799. PMID: 20031688

[30] Neves MI Moroni L Barrias CC . Modulating alginate hydrogels for improved biological performance as cellular 3D microenvironments. Front Bioeng Biotechnol. (2020) 8:665. doi: 10.3389/fbioe.2020.00665. PMID: 32695759 PMC7338591

[31] Lee KY Mooney DJ . Alginate: properties and biomedical applications. Prog Polym Sci. (2012) 37:106–26. doi: 10.1016/j.progpolymsci.2011.06.003. PMID: 22125349 PMC3223967

[32] Yao L Rathnakar BH Kwon HR Sakashita H Kim JH Rackley A . Temporal control of PDGFRα regulates the fibroblast-to-myofibroblast transition in wound healing. Cell Rep. (2022) 40:111192. doi: 10.1016/j.celrep.2022.111192. PMID: 35977484 PMC9423027

[33] Nemeth J Schundner A Quast K Winkelmann VE Frick M . A novel fibroblast reporter cell line for *in vitro* studies of pulmonary fibrosis. Front Physiol. (2020) 11:567675. doi: 10.3389/fphys.2020.567675. PMID: 33162897 PMC7582034

[34] Roebuck KA Finnegan A . Regulation of intercellular adhesion molecule-1 (CD54) gene expression. J Leukocyte Biol. (1999) 66:876–88. doi: 10.1002/jlb.66.6.876. PMID: 10614768

[35] Hynes RO . Integrins: versatility, modulation, and signaling in cell adhesion. Cell. (1992) 69:11–25. doi: 10.1016/0092-8674(92)90115-s. PMID: 1555235

[36] Ezzo M Spindler K Wang JB Lee D Pecoraro G Cowen J . Acute contact with profibrotic macrophages mechanically activates fibroblasts via αvβ3 integrin-mediated engagement of Piezo1. Sci Adv. (2024) 10:eadp4726. doi: 10.1126/sciadv.adp4726. PMID: 39441936 PMC11498225

[37] Heikamp EB Patel CH Collins S Waickman A Oh M-H Sun I-H . SUPP - The AGC kinase SGK1 regulates TH1 and TH2 differentiation downstream of the mTORC2 complex. Nat Immunol. (2014) 15:457–64. doi: 10.1038/ni.2867. PMID: 24705297 PMC4267697

[38] Wiesolek HL Bui TM Lee JJ Dalal P Finkielsztein A Batra A . Intercellular adhesion molecule 1 functions as an efferocytosis receptor in inflammatory macrophages. Am J Pathol. (2020) 190:874–85. doi: 10.1016/j.ajpath.2019.12.006. PMID: 32035057 PMC7180595

[39] Bernatchez SF Atkinson MR Parks PJ . Expression of intercellular adhesion molecule-1 on macrophages *in vitro* as a marker of activation. Biomaterials. (1997) 18:1371–8. doi: 10.1016/s0142-9612(97)00072-0. PMID: 9363337

[40] Bui TM Wiesolek HL Sumagin R . ICAM-1: A master regulator of cellular responses in inflammation, injury resolution, and tumorigenesis. J Leukoc Biol. (2020) 108:787–99. doi: 10.1002/JLB.2MR0220-549R. PMID: 32182390 PMC7977775

[41] Gu W Yao L Li L Zhang J Place AT Minshall RD . ICAM-1 regulates macrophage polarization by suppressing MCP-1 expression via miR-124 upregulation. Oncotarget. (2017) 8:111882–901. doi: 10.18632/oncotarget.22948. PMID: 29340098 PMC5762366

[42] Goswami R Arya RK Sharma S Dutta B Stamov DR Zhu X . Mechanosensing by TRPV4 mediates stiffness-induced foreign body response and giant cell formation. Sci Signal. (2021) 14:eabd4077. doi: 10.1126/scisignal.abd4077. PMID: 34726952 PMC9976933

[43] Liu H Wu M Jia Y Niu L Huang G Xu F . Control of fibroblast shape in sequentially formed 3D hybrid hydrogels regulates cellular responses to microenvironmental cues. NPG Asia Mater. (2020) 12:45. doi: 10.1038/s41427-020-0226-7. PMID: 37880705

[44] Li X Liu Y Tang Y Xia Z . Transformation of macrophages into myofibroblasts in fibrosis-related diseases: emerging biological concepts and potential mechanism. Front Immunol. (2024) 15:1474688. doi: 10.3389/fimmu.2024.1474688. PMID: 39386212 PMC11461261

[45] Chen B Li R Kubota A Alex L Frangogiannis NG . Identification of macrophages in normal and injured mouse tissues using reporter lines and antibodies. Sci Rep. (2022) 12:4542. doi: 10.1038/s41598-022-08278-x. PMID: 35296717 PMC8927419

