# Supplementary material for: Co-culture with fibroblasts in stiff 3D scaffolds increases CD54 and CD140a expression on macrophages
Source: Front Immunol. 2026 Jun 3;17:1771248. doi: 10.3389/fimmu.2026.1771248 (PMC13272094; doi:10.3389/fimmu.2026.1771248)
Supplement: Supplementary file 1 [file DataSheet1.pdf]

frontiers

SUPPLEMENTARY INFORMATION

Co-culture with fibroblasts in stiff 3D scaffolds increases CD54 and CD140a expression on macrophages

Jennessa WX Ng[1,2], Santosh TRB Rao[3], Emily H Field[4,5], Kaitlyn Ritchie[1,2], Mark D Wright[6], Nicholas P Reynolds[1,4,5], Sean W Cutter[1,2,8], Katrina J Binger[1,2,7,8*]

[1]Department of Biochemistry & Chemistry, School of Agriculture, Biomedicine and Environment, La Trobe University, Melbourne, Victoria 3086, Australia.

[2]Centre for Cardiovascular Biology & Disease Research, La Trobe Institute for Molecular Science (LIMS), La Trobe University, Melbourne, Victoria 3086, Australia

[3]Holsworth Biomedical Research Centre, Department of Rural Clinical Sciences, La Trobe Rural Health School, La Trobe University, Bendigo, Victoria 3550, Australia

[4]Department of Biology, School of Science, RMIT University, Melbourne, Victoria 3082, Australia

[5]Aikenhead Centre for Medical Discovery, Fitzroy, Victoria 3065, Australia

[6]Department of Immunology & Pathology, Alfred Medical Research and Education Precinct, School of Translational Medicine, Monash University, Melbourne, Victoria 3004, Australia.

[7]Department of Biochemistry & Molecular Biology, Biomedicine Discovery Institute, Monash University, Clayton, Victoria 3800, Australia.

[8]equal contribution

***Correspondence:**
Katrina Binger
katrina.binger@monash.edu

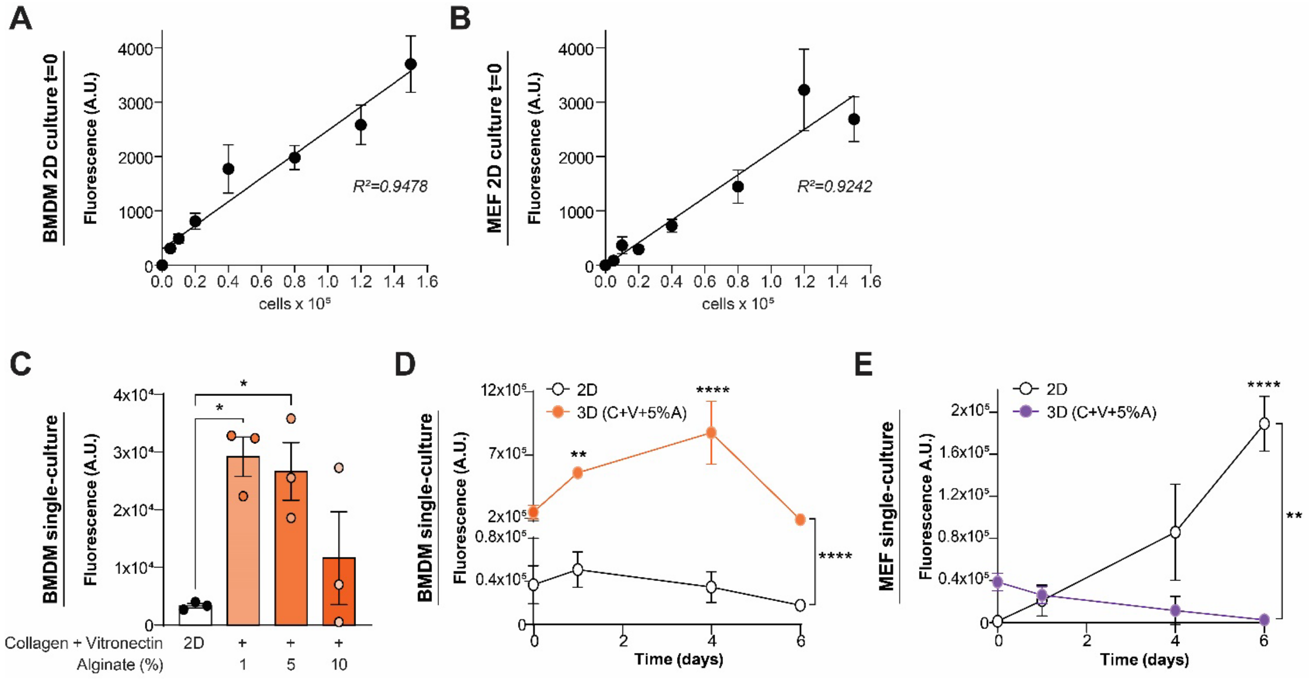

Supplementary Figure 1. BMDMs and MEFs have increased absolute alamarBlue fluorescence upon 3D culture. A-B) To ensure the linearity of the alamarBlue assay in 2D, increasing cell numbers of BMDMs and MEFs were seeded on traditional 2D plasticware and allowed to attach for 2 h before the addition of alamarBlue. Absolute fluorescence readings are shown, corrected by subtraction from cell-free wells to remove background signal. **C)** BMDMs were seeded in 3D collagen-vitronectin scaffolds with increasing concentrations of alginate. Traditional 2D plasticware coated with a monolayer of collagen was compared as a control. Cells were cultured for 24 h before addition of alamarBlue. Absolute fluorescence readings are shown, corrected by subtraction from respective cell-free 3D scaffolds or 2D wells to remove background signal. **D-E)** BMDMs and MEFs were cultured separately in 3D collagen-vitronectin-5% alginate scaffolds or 2D controls for 6 days. Absolute fluorescence values are shown after background correction as in A. Data in C-E are the pooled means of at least 3 independent experiments where individual data points are shown in D. Error bars show SEM. Significance in C was tested by one-way ANOVA; D-E by two-way ANOVA with post hoc analyses. Significance is indicated as *$p<0.05$, **$p<0.01$, ****$p<0.0001$.

This is a provisional file, not the final typeset article

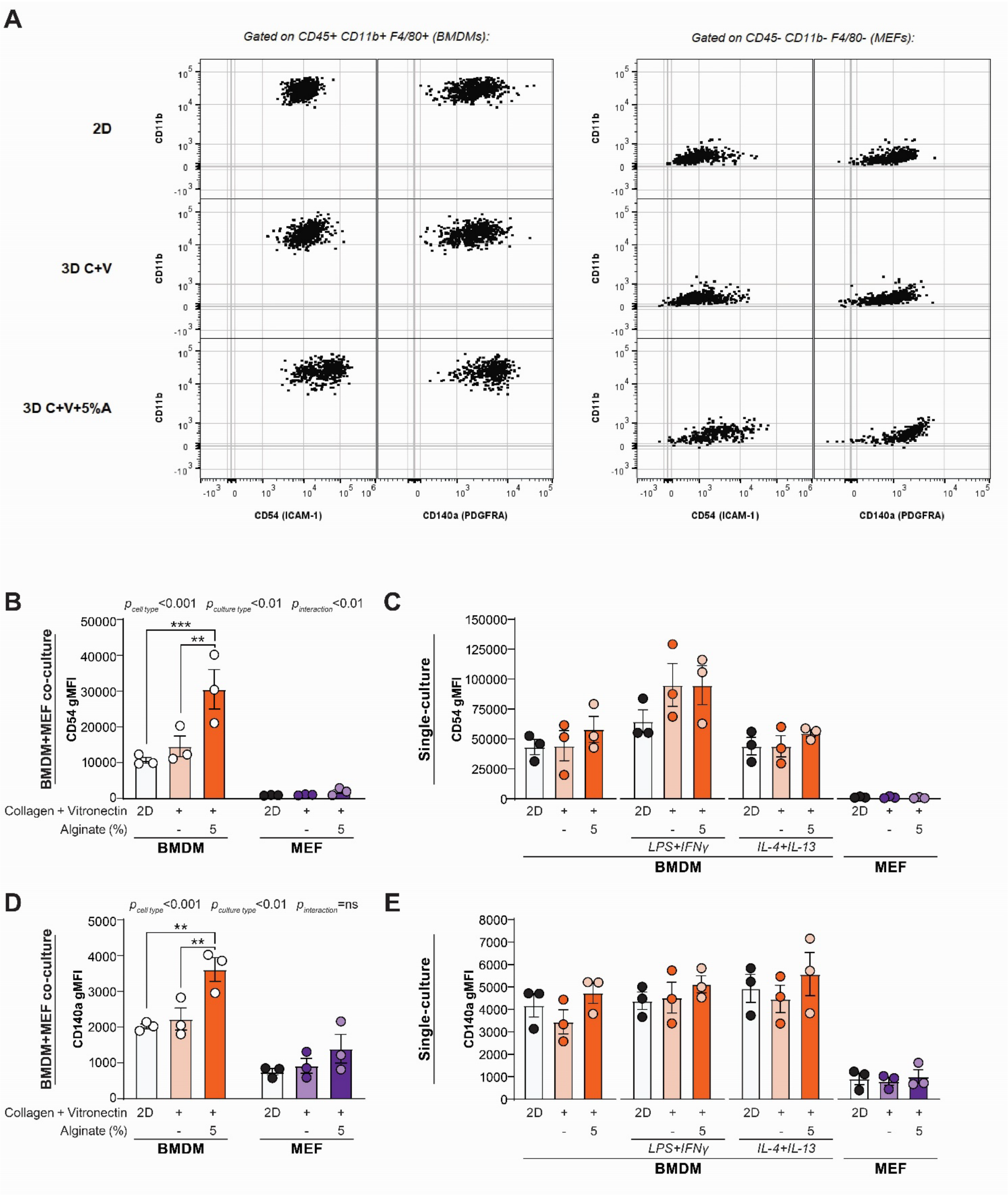

Supplementary Figure 2. Representative flow cytometry dot plots and absolute mean fluorescence intensity of CD54 and CD140a expression on BMDMs and MEFs. A) Flow cytometry analysis evaluating the surface expression on BMDMs and MEFs following 24 h of co-culture. Representative dot plots show the expression profiles of CD54 (ICAM-1) and CD140a (PDGFRA) on BMDMs ($CD45^{+}$ $CD11b^{+}$ $F4/80^{+}$; left panels) and MEFs ($CD45^{-}$ $CD11b^{-}$ $F4/80^{-}$)

cultured in 2D, 3D collagen-vitronectin (C+V) or 3D collagen-vitronectin-5% alginate (C+V+5%A) scaffolds. **B)** Geometric mean fluorescence intensity (gMFI) of CD54 (B-C) and CD140a (D-E) on BMDMs and MEFs after co-culture or single-culture in the indicated models. Data show the pooled means of 3 independent experiments. Errors are SEM. Data in B-E was tested by two-way ANOVA with post-hoc analyses. Significance is indicated as **p<0.01, ***p<0.001.

This is a provisional file, not the final typeset article
